# Perinatal palliative care: challenges and priorities in comprehensive nursing care

**DOI:** 10.15649/cuidarte.5415

**Published:** 2026-03-04

**Authors:** Damaris Angélica Palomino-Alzate, Jhon Edinson Basto-Rueda, Kenia Yoelis Martínez-Torres, Javier Mauricio Sánchez-Rodríguez, German Andrey Sarmiento-Velasco

**Affiliations:** 1 Egresada Especialización en Enfermería en Cuidados Paliativos, Fundación Universitaria Sanitas. Bogotá, Colombia. E-mail: dapalominoal@unisanitas.edu.co Fundación Universitaria Sanitas Bogotá Colombia dapalominoal@unisanitas.edu.co; 2 Egresado Especialización en Enfermería en Cuidados Paliativos, Fundación Universitaria Sanitas. Bogotá, Colombia. E-mail: je.bastoru@unisanitas.edu.co Fundación Universitaria Sanitas Bogotá Colombia je.bastoru@unisanitas.edu.co; 3 Egresada Especialización en Enfermería en Cuidados Paliativos, Fundación Universitaria Sanitas. Bogotá, Colombia. E-mail: ky.martinezto@unisanitas.edu.co Fundación Universitaria Sanitas Bogotá Colombia ky.martinezto@unisanitas.edu.co; 4 Docente Universidad Autónoma de Bucaramanga. Bucaramanga, Colombia. E-mail: jsanchez477@unab.edu.co Universidad Autónoma de Bucaramanga Bucaramanga Colombia jsanchez477@unab.edu.co; 5 Facultad de Enfermería, Fundación Universitaria Sanitas. Bogotá, Colombia. E-mail: gasarmientove@unisanitas.edu.co Fundación Universitaria Sanitas Bogotá Colombia gasarmientove@unisanitas.edu.co

 Dear Editor:

 Nowadays, when we talk about pregnancy, labor, childbirth, or the arrival of a new family member, it is often done with feelings of joy and life. However, within the field of maternal and neonatal care, many fetuses and newborns face the risk of death in the days, months, or years that follow birth^1^.

 In this regard, according to the meta-synthesis conducted by Kuforiji et al.^2^ in the United Kingdom, one of the main problems arising from perinatal death, from the perspectives of women who have experienced it, is the lack of understanding and support from healthcare professionals and the community. This issue is because, in many cases, women feel guilty and experience negative emotions, which can be exacerbated by the lack of adequate medical explanations regarding the causes of perinatal death. Furthermore, negative responses from family members and the community, based on cultural beliefs, also contribute to this lack of support.

 In situations or scenarios such as these, Cacciatore and Thieleman^3^ argue that the death of a newborn represents a complex series of hormonal changes in the mother, giving rise to an intricate emotional, physiological, and spiritual experience that triggers feelings of pain and shame. However, in most cases, this tragedy is overlooked, and the necessary attention is not provided by one or more actors involved, either by minimizing the unborn child’s life or the parents’ feelings. This series of events initiates a grieving process that may be disrupted or distorted by the aforementioned factors, which, if left unaddressed, can lead to deep and lasting emotional wounds.

 Additionally, research conducted by Fernández-Sola et al.^4^ emphasizes that perinatal loss affects not only the parents (mother and father) but also the nuclear and extended family in general, depending on the family dynamics of the group of individuals involved. Such an effect will also be noticeable in daily dynamics and the roles individuals play within their sociocultural context. These results align with the findings reported by Martínez-Esquivel et al.^5^, who observed that these processes can lead to complicated grief due to the persistence of bonds.

 This situation produces a series of effects that directly impact the relationships among these groups. In this sense, support during these processes becomes a key element in the attention and care provided, regardless of whether or not the individuals involved are significantly affected by the neonatal loss. For this reason, it is necessary to integrate both the healthcare team and the available community or support networks in these situations^4^,^6^.

 Therefore, perinatal palliative care acquires great importance, as its implementation represents a differential factor in achieving the objective of preventing, alleviating, and intervening in the physical, psychological, and social suffering of fetuses or newborns diagnosed with a life-limiting disease and categorized as individuals in the end-of-life phase. This differential factor is accompanied by monitoring and support for the immediate and extended family, tailored to their needs^7^,^8^.

 Chavarro et al.^8^ regard pediatric palliative care as a specialty that has evolved over the years and has been incorporated into the health systems of different countries through legislative processes and the creation of associations involving multiple actors (health professionals and family members of patients eligible for palliative care), who aim to improve access to health services in the earlier stages of the disease, advance research, have better-trained human resources for health, and reduce the barriers perpetuated by ignorance, myths, and prejudices that exist in the society in general^8^.

 According to Santos et al.^9^, in 2023, it was analyzed that, thanks to technological advances have improved early detection and survival rates for complex chronic diseases in the pediatric population, which has increased the need for palliative care aimed at alleviating suffering and improving quality of life. These findings refute the belief that palliative care is not designed for children with chronic health conditions. Although infant mortality has decreased and life expectancy has increased in several regions worldwide, the need for support from interdisciplinary pediatric palliative care teams at different levels of healthcare is becoming increasingly frequent. In the specific case of perinatal palliative care, such support begins before birth.

 Furthermore, the National Institute of Health of Colombia indicated that, by 2022, congenital malformations and gestational hypertensive disorders had become leading causes of mortality in developed countries. Taking this into account, one of the Millennium Development Goals that result in the Sustainable Development Goals is to ensure women's full access to all birth control services, as well as to the information and education they require, and to end preventable deaths among newborns^10^.

 The systematic review conducted by Moreno-Tirado et al.^11^ in 2023 suggests that, although clinical practice guidelines exist to guide the actions of physicians and nurses, perinatal palliative care remains overshadowed by a lack of knowledge and training tools. This situation makes it imperative for nurses to receive specific education integrating both theory and practice.

 Additionally, the existing literature remains limited regarding the different biopsychosocial issues associated with the need for care and emotional burden experienced by parents. These include the management of sensitive and confidential information, supporting parental decision-making, accompanying parents at the time of their newborn’s death, providing follow-up to these families, facilitating participation in self-help groups, and offering religious or spiritual guidance. It is important to emphasize that nursing care should not only focus on the newborn who is dying or has already passed away, but also encompass the entire family system, safeguarding the parents’ dignity and ensuring their physical, psychosocial, emotional, and spiritual health^12^.

 Thus, the discipline of human care, through the different existing nursing theories, provides tools to guide the care process. One example is Swanson's Theory of Caring, whose objective is the dual participation of professionals and families, enabling care to be provided in accordance with parental needs. This theoretical approach aligns with the findings of Nurse-Clarke et al.^12^ regarding the grieving process, in which they note that emotional burdens are experienced not only by parents but also by nurses, directly influencing the current and previous experiences of perinatal death among parents.

 In many cases, the identification of needs observed in clinical practice has been addressed empirically. From these, we can highlight the need to respect and facilitate diverse cultural farewell rituals, understood as a form of respect for emotions and silences. Similarly, providing care for the deceased newborn, such as allowing the parents to name the child, giving them the identification bracelet, and other keepsakes, allows them to construct a memory of their lost child. However, there are few instruments available to assess these needs^11^.

 According to a study by Cruz et al.^13^ in 2019, another important aspect involves addressing and containing the feelings of parents who have lost a child, as well as their prospects of a future child or adolescent, highlighting the importance of the healthcare team’s support in clarifying doubts and concerns. This study also mentions the indifference in some Intensive Care Units, where different types of monitoring used prevent parents from hugging their children, leaving them with a physical, spiritual, and emotional void.

 Furthermore, regarding the role of the healthcare team, and particularly nursing, research conducted by Fernández-Férez et al.^14^ identifies a persistent need for professionals equipped with specialized knowledge and trained in emotional support and containment. These competencies and skills are fundamental to support an adequate grieving process, inviting parents to engage in the different religious, spiritual, cultural, or mourning practices they consider relevant as part of that process, all in the light of empathy and warmth, essential characteristics of compassion.

 The aforementioned authors also highlight that nursing has contributed less research on pediatric or perinatal palliative care than other health professions^15^. This finding highlights a gap in nurses’ knowledge regarding the care of populations with specific needs in perinatal palliative care. In addition, Ravaldi et al.^16^ show that the main barriers to providing such care may be related to a lack of resources and inadequate theoretical and practical training in this field.

 In the Colombian context, Sánchez Cárdenas et al.^17^ note that, although progress has been made in developing regulations for palliative care services and increasing the number of trained personnel, only a few undergraduate and graduate nursing and medical programs include content in their curricula or are specific programs in this area. Furthermore, it remains unclear whether the pediatric population has been included in these educational processes.

 Therefore, nurses must promote practice-based research aimed at developing strategies and tools to improve the comprehensive approach to families facing perinatal loss. Along the same lines, education is required to build both knowledge and confidence in abilities, as well as to encourage the recording of their activities^16^. In addition, strengthening perinatal palliative care enables the timely identification of needs, reduces the emotional burden on healthcare personnel, and ensures adequate support^15^,^18^,^19^.

 In such situations, nurses should foster the rigorous documentation of interventions and encourage research that highlights their role in perinatal palliative care, thereby systematizing the care process and raising awareness among healthcare staff. This approach contributes to more structured and effective care, benefiting patients, families, and communities, as well as the profession's own knowledge and its visibility^17^,^18^.

 Based on the foregoing, this paper aims to create value by addressing the profound and often hushed-up needs of fetuses, newborns, and their families when life is threatened from the outset. In addition to requiring specialized clinical care, these circumstances call for a warm, ethical, and humane presence from the healthcare team, particularly from nurses.

 Likewise, it aims to promote understanding that these experiences contribute not only to strengthening training, care management, research, and daily practice programs but also to promoting comprehensive and respectful care that upholds the vulnerability and dignity of life in all its forms.

 As healthcare professionals, we cannot deny that these realities exist. It is the responsibility of nursing, as both a scientific and human discipline, to lead the creation of perinatal palliative care models that respond in a timely, respectful, and sensitive manner to those who need them most, because even in the most fragile moments, caring remains a true expression of love and justice.
